# Large-area OLED substrate printing path planning method based on multi-head GAT imitation learning to solve partitioned integer programming

**DOI:** 10.1038/s41598-025-08355-x

**Published:** 2025-07-01

**Authors:** Jiacong Xiong, Jiankui Chen, Yiqun Li, Xiao Yue, Yu Fu, Zhouping Yin

**Affiliations:** 1 The State Key Laboratory of Intelligent Manufacturing Equipment and Technology, School of Mechanical Science and Engineering, Huazhong University of Science and Technology, Wuhan, 430074 China; 2 Wuhan National Innovation Technology Photoelectric Equipment Co., LTD., Wuhan, 430074 China

**Keywords:** Printing display, Substrate angular deflection, Integer programming, Parallel modelling, Multi-head graph attention network, Electrical and electronic engineering, Mechanical engineering, Computer science

## Abstract

Inkjet printing is considered a very promising technology in the field of organic light-emitting diode (OLED) substrate manufacturing. Compared with the vapor deposition process, inkjet printing has the advantages of a simple process, high material utilization and applicability to a wide range of display manufacturing processes. However, during the inkjet manufacturing process, the resolution of the printhead (nozzle per inch, NPI) usually does not match the pixel resolution of the substrate (pixel per inch, PPI). Therefore, the travel path of the printhead module must be planned to minimize the number of print cycles required to complete the pattern. This involves a challenging multi-objective optimization process. The difficulty intensifies in large-area OLED production, where angular alignment errors in the substrate are magnified. This results in an exponential increase in pixels requiring planning, with the total pixel pit count reaching hundreds of millions. In addition, the time complexity of the planning problem grows exponentially, denoted as O(m^n^), and the space complexity grows rapidly with the matrix dimension. This problem is NP-hard. This problem has a significant impact on the productivity of the manufacturing process. In this paper, a large-area substrate printing planning algorithm based on graph attention networks and integer programming (GIP-LASP) is established. GIP-LASP provides partitioning rules and parallel modeling methods specifically for substrate misalignment angles, and proposes imitation learning based on a multi-head graph attention network on a SCIP solver, which is applied to the solution of the printing planning problem. The planning method was implemented on a G4.5 half-size substrate with a resolution of 394 PPI, and the color filter (CF) layer was successfully printed.

## Introduction

Organic light-emitting diodes (OLEDs) consist of multiple layers of functional and luminescent materials. Using inkjet printing technology, solutions corresponding to each functional layer can be precisely deposited as droplets into the pixel pits^1^. After drying and curing, a thin film is formed, ultimately enabling the encapsulation of the device for display purposes. Inkjet printing utilizes a non-contact, maskless process that effectively prevents the waste of functional materials. The display material is precisely deposited into the corresponding pixel pits as needed, achieving up to 90% material utilization^2^, which significantly reduces production costs^3^. Production efficiency is further enhanced by the synergistic operation of multiple nozzles. It is foreseeable that inkjet printing technology will gradually become the mainstream in the display field^4^. However, it still faces significant challenges in terms of precision and efficiency, especially in applications involving large-area, high-resolution display substrates^5^.

The number of pixel pits on a large-area, high-resolution substrate can reach into the billions. Due to limitations in printhead manufacturing accuracy, the volume of droplets ejected from each printhead may vary. To ensure uniform deposition across all pixel pits on the display device, droplets of different volumes must be mixed to achieve consistent filling.

**Fig. 1:**
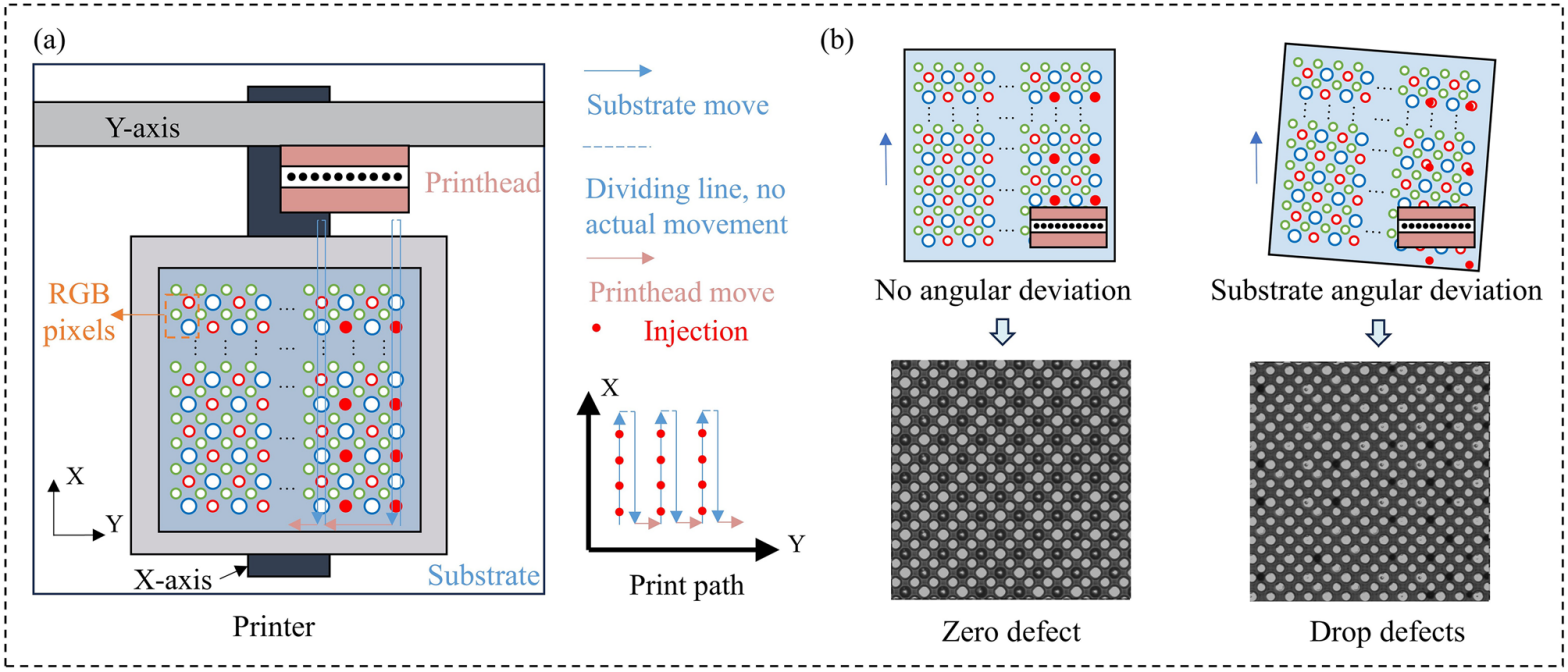
The fundamental structure of inkjet printing display equipment and the dimensionality catastrophe in printing planning caused by substrate misalignment: (**a**) the primary components participating in printing are the printhead and substrate, housing nozzle arrays and pixel pit arrays, respectively, the printing path during the inkjet printing process, requiring design through printing planning; (**b**) substrate angular deviation causes the drop defects.

The main components of the printing device include a printhead, a substrate stage, and a visual localization camera. As shown in Fig. 1a, the nozzles are arranged on the printhead in a fixed-pitch array, while pixel pits on the substrate are arranged at regular intervals. For large-area substrates, the total number of pixel pits can reach the billions. Figure 1a provides a brief overview of the printhead’s motion path during printing. In Fig. 1b, we observe that when there is no angular deviation in the substrate, pixel pits aligned along the X-axis are projected as a single column within the field of view, significantly reducing the number of pixel pits involved in print planning. However, in practice, slight angular deviations often occur during substrate placement. These small deviations can amplify dimensional mismatches across the substrate, making projection-based simplification infeasible and ultimately leading to a dimensional catastrophe in the planning process.

Madigan^6^ proposed both intra-pixel and inter-pixel mixing methods. The intra-pixel mixing method independently considers the homogeneity of a single row of pixel pits along the print path and ensures that the volume of the mixed solution remains within an acceptable error margin. However, it does not account for the scaling effects caused by large-scale pixel pit arrays. In contrast, the inter-pixel mixing method ensures that each row of pixel pits meets the volume deviation requirement while comprehensively considering the volume uniformity of deposited droplets across the entire substrate. Decision variables in the droplet mixing process include the movement of the printhead and the scheduling of nozzle jets. Due to the vast number of pixels on a high-resolution, large-area substrate and the large number of nozzles involved in printing^7^, the resulting droplet mixing model exhibits extremely high dimensionality in both constraints and decision variables. Print planning is essentially a multi-objective optimization problem involving the spatial matching between nozzles and pixel positions. Angular deviations result in all pixels being involved in the planning process, which leads to an exponential increase in planning time and memory consumption as the matrix dimension grows. This renders the planning process an NP problem, with a time complexity of O(m^n^), where m is the number of possible integer values and n is the number of integer variables. This significantly impacts the efficiency of inkjet print planning and prolongs the overall printing time.

Currently, major industry leaders have made significant progress in planning algorithms for display device printing, although these results have not been publicly disclosed. For instance, Yue^8^ investigated the effect of single-pulse waveform modulation on droplet jetting, aiming to reduce droplet volume variation during the printing process. Hong^9^ introduced a kinetic model to analyse the merging behaviour of droplets impacting rectangular pixels, showing that lower initial droplet velocity and smaller positioning errors contribute to more reliable OLED inkjet printing. Gao^10^ analysed skewed droplet jetting from nozzles and compared the results with those of normal nozzles to determine drop deviations. Abnormal nozzles were deactivated during printing to ensure quality. Zhang^11^ proposed a method based on Coherent Scanning Interferometry (CSI) for accurately measuring deposited droplets, which was applied to assess droplet placement in printed pixel pits. Merklein^12^ explored the effect of different printing methods on the uniformity of display performance using a fixed dot spacing. While these printing algorithms are either undisclosed or applied to TFE layer printing, and they do not require sophisticated algorithms to address nozzle anomalies, merely meeting the demand for a sufficient number of qualified nozzles on the print head. We need to explore pixel-filling printing for RGB, such as the intelligent blending model proposed by Madigan^6^, and more effective algorithms for larger dimensions.

Other researchers have also proposed their own algorithms or ideas. Moro^13^ proposed solving the problem of inhomogeneous “Mura” in displays by introducing randomization or employing more complex algorithms. Phung^14^ designed a coded processing unit and a vector printing algorithm to plan the X-axis and Y-axis printing motions, as well as nozzle ejection actions, by generating droplets at equally spaced intervals. This approach helps prevent uneven line widths at the beginning and end of inline printing. Chiu^15^ proposed a bitmap print image cropping method with conceptual compensation, which adjusts the ignition pulse according to substrate expansion caused by heat or stress. Lin^16^ proposed a novel printing algorithm based on printhead rotation and a staggered printing method for OLED display manufacturing. Kim^17^ proposed an algorithm to redefine the resolution of the printed image and plan the printing process in bitmap mode. Li^18^ proposed an optimized waveform design to quickly eliminate residual oscillations in EHD DOD printing by adding extra voltage pulses at jet stop and retraction. Chang^19^ proposed an interleaved rotation algorithm for planning the print path and nozzle protrusion actions. The algorithm adjusts the number of nozzles per inch (NPI) by rotating the printhead angle. Wang^20–22^ modelled the print planning process as an integer planning problem and its variants, with the objective of minimizing the number of prints to achieve optimal planning for a 200 × 200 mm pixel-pitted substrate. Yang^23^ proposed an optimized nozzle design for a multi-nozzle EHD printhead to achieve large-scale, crosstalk-free printing. Lian^24^ applied deep reinforcement learning to OLED printing to achieve multi-objective optimization, although the method was not experimentally verified.

Existing researchers have also made progress in the study of large-area inkjet-printed substrates. Kang^25^ discussed the challenges in mass-producing printed display devices and demonstrated an 18.2-inch, 202 pixel per inch (PPI) OLED panel. Zhou^26^ developed halogen-free inks and successfully achieved large-area patterned inkjet printing on a flexible substrate with efficient blue light emission (200 × 200 mm). Kwon^27^ fabricated an organic/inorganic hybrid TFE by combining PEALD device modules and IJP device modules within a cluster of OLED fabrication equipment, resulting in a large-area package structure measuring 100 × 100 mm. Kant^28^ developed a handheld large-area panel (LAiP) substrate (120 × 120 mm) with an effective area of 80 × 80 mm, realized using square chrome grid lines. Subsequently, Kant^29^ successfully fabricated an 80 × 80 mm OLED using a small molecule phosphorescent dopant. In addition, he^30^ produced a 120 × 120 mm OLED sign with a minimum opening size of 18 μm using a single-step patterned complex design.

Print planning problems for display manufacturing can be formulated as combinatorial optimization problems. In recent years, the development of artificial intelligence techniques has influenced many industries, including the direction of optimization problem-solving. However, optimization problems do not exhibit an obvious Euclidean spatial structure. Unlike images or text data, changes in the order of variables in the input to a neural network should not be interpreted as meaningful features of the optimization problem. The emergence of graph neural networks (GNNs) addresses this issue by representing the variables and constraints of an optimization problem as nodes in a graph. Edges are constructed based on the relationships among variables, thereby preserving the spatial structure between nodes. For example, Gasse^31^ formulated the integer programming problem as a bipartite graph and used graph convolutional network (GCN) imitation learning to mimic the SCIP solver, surpassing its original solving efficiency. Wu^32^ applied GNNs to learn approximation rules for the goal space decomposition algorithm in solving multi-objective integer programming problems. Nair^33^ proposed a hybrid solving framework based on Gasse’s neural diving and neural branching architecture, which significantly outperforms the original SCIP solver and further reduces the optimality gap. Gupta^34^ proposed a hybrid framework where a graph convolutional neural network is applied only at the root node of the branching decision tree to reduce computational cost. Ding^35^ constructed the integer programming problem as a tripartite graph, with goal nodes represented as individual nodes, although their method was limited to solving 0–1 programming problems. The application of GNNs to solving integer programming problems is rapidly developing and has demonstrated effectiveness in various aspects.

In summary, existing studies have not disclosed pixel pit filling algorithms for inkjet printing on large-size substrates. This is primarily because, compared to TFE printing, pixel pit printing requires avoiding deposition outside the target pixel pit or onto the wrong one, and demands inkjet landing accuracy within 5 μm. The angular deviation inherent in large-size substrates further increases the difficulty of achieving defect-free inkjet printing. Meanwhile, the application of artificial intelligence techniques to optimization problems in this context presents broader opportunities for advancement. In this paper, we propose a large-area substrate printing planning algorithm based on graph attention networks and integer programming (GIP-LASP). The method consists of (1) an inkjet integer planning model based on parallel modelling of substrate partitioning, and (2) an attention network algorithm for multi-head maps. We analyse the planning process of large-pixel panels through parametric modelling and implement substrate partitioning based on substrate angular deviation. To realize the model solving, a solving algorithm based on graph attention network imitation learning is designed. It achieves more efficient solving by imitating the branching strategy of the solver. At the same time, a parallel planning method is employed to generate print planning data in order to address the challenges of printing large-size substrates in manufacturing.

The main contributions of this paper are as follows: (1) An angular deviation-based printing direction partitioning method is proposed, which significantly reduces the number of pixels involved in printing. (2) A multi-parameter computational simulation is conducted for the printhead arrangement direction, resulting in the optimal partitioning intervals and stopping point spacing ranges. (3) An integer programming solution algorithm based on multiple Graph Attention Networks (GATs) is developed for printhead display planning, which outperforms the original SCIP solver in terms of solution efficiency. (4) Defect-free printing on a 394 PPI G4.5 half-size substrate is achieved using the proposed scheme, validating its effectiveness. The structure of this paper is as follows: Sect. 1 introduces the advantages and planning challenges of printing displays on large-size substrates. Section 2 provides a comprehensive review of related research work. Section 3 presents the design of an inkjet printing integer planning method based on parallel modelling of substrate partitions, as well as an integer programming solver based on Graph Attention Networks. Sections 4 and 5 present the simulation and experimental results of the proposed method. Section 6 summarizes the paper and discusses potential directions for future research.

## Design of a parallel planning methodology for large-scale substrate partitioning

**Fig. 2:**
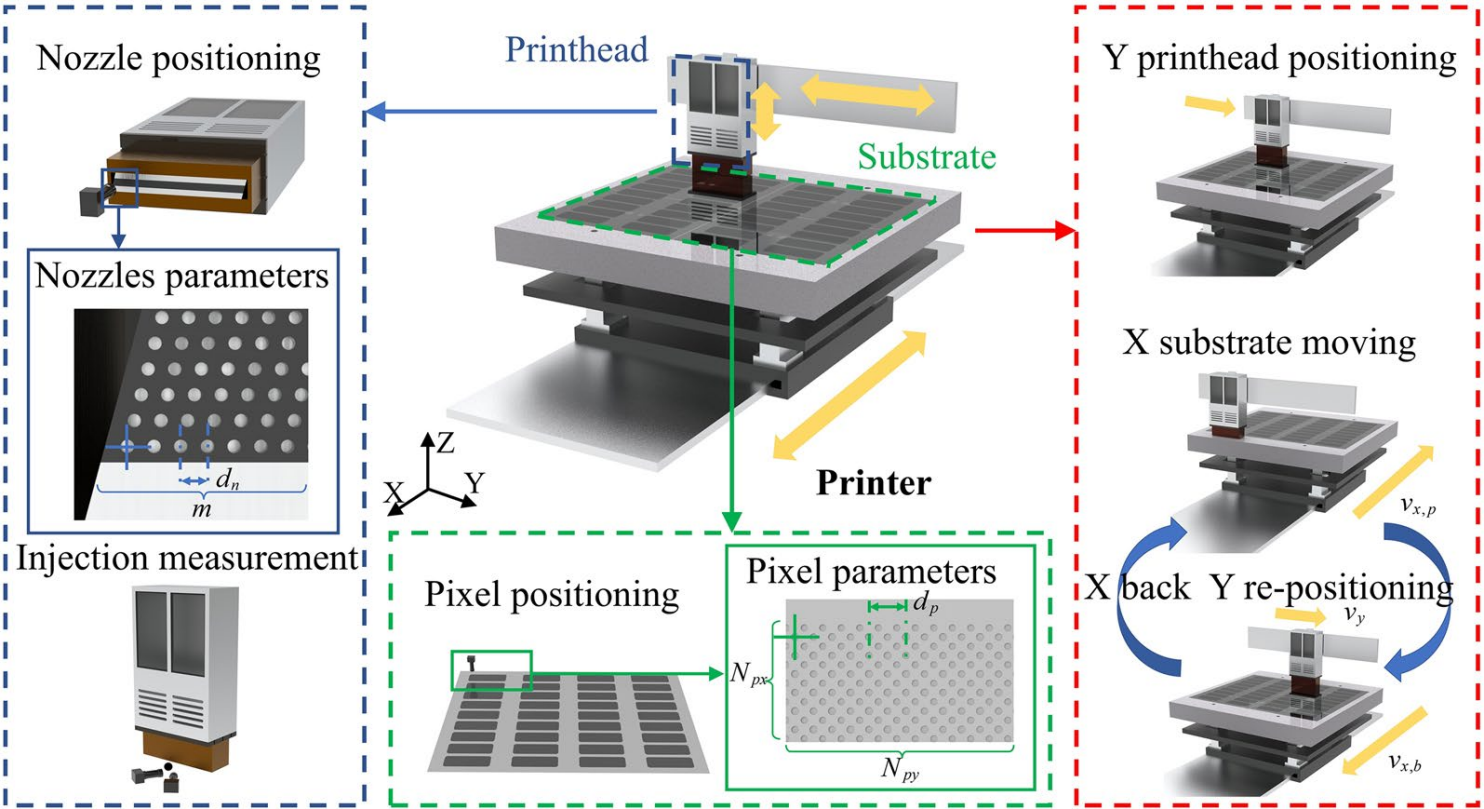
Key components of printing equipment related to print planning and the movement of printhead modules and substrate platforms during print execution.

In this study, the design of the print planning method is based on the general principles of current printing equipment development. The two main components involved in printhead planning are the printhead module and the substrate motion platform, as shown in Fig. 2. The printhead module is mounted on the Y- and Z-axes to enable positioning in the Y-direction and height adjustment along the Z-direction. The substrate motion platform is mounted on the X-axis, and its movement triggers the operation of the printhead during the printing process. The current position of the motion platform is monitored using a linear scale. The entire printing system is enclosed within a glove box to maintain stable pressure, temperature, humidity, and other environmental factors, providing a robust technical foundation for high-precision printing.

To enable printing on large-area substrates, multiple printheads are integrated within the printhead module to increase the effective print width. Each printhead features an array of nozzles, whose arrangements vary depending on the manufacturer—typically in either linear or staggered matrix configurations. The staggered matrix layout is designed to achieve a higher NPI density. Although the nozzles are not aligned on a single line, their jetting timing can be algorithmically adjusted via software control. By measuring the coordinates of reference nozzle, the full coordinate set of all nozzles can be derived, along with nozzle-to-nozzle spacing. The positional deviation of each nozzle is characterized through test printing, and compensation is applied accordingly. Additionally, the droplet volume for each nozzle is measured using a drop observation system. Since the printheads are mounted at an offset angle, the true nozzle coordinate matrix can be calculated by incorporating the angular offset into the positioning model. In this study, the printhead module is mathematically abstracted and digitized using four key parameters: (1) printhead angle, (2) nozzle coordinate matrix, (3) nozzle drop deviation matrix, and (4) nozzle volume matrix. This abstraction improves the generalizability of the model.

In a simplified view, pixel pit substrates can also be regarded as arrays of pixel pits arranged in a defined pattern, which can be similarly parameterized. A large-area substrate typically contains multiple display panels, which are printed and later diced into individual units, such as smartphone screens. Each panel features RGB pixel pits arranged at fixed intervals. These pixel pits may vary in shape and layout, and similar to the staggered arrangement of nozzles, pixel pit arrays can also be intentionally misaligned to achieve higher PPI densities. As such, the pixel pits targeted in this study are not fundamentally different from conventional oblong-shaped RGB pixel pit configurations. The ultimate goal is to determine the coordinates of all pixel pits on the substrate and the corresponding droplet volume required for each. Therefore, in this work, the pixel pit substrate is modelled using four key parameters: (1) substrate declination angle, (2) pixel coordinate matrix, (3) required print volume per pixel, and (4) pixel pit size.

By parameterizing both the printhead module and the pixel pit substrate, we can establish the spatial relationship between all nozzles and the pixel pits, laying the foundation for subsequent print path planning. Before this, it is essential to outline the printing process in display manufacturing. Once the unprinted substrate is transferred from the assembly line to the printing equipment, it undergoes alignment and correction to determine its position and angular offset. A parameterized printing recipe is then loaded for planning and computation, followed by the execution of the printing process. As illustrated in Fig. 2, the printing process comprises the following steps: (1) The printhead module moves to the designated Y-axis position. (2) The corresponding print data for the current pass is retrieved. (3) The substrate motion platform moves along the X-axis, triggering the jetting action. (4) The substrate platform resets to the initial position. These steps are repeated until the entire substrate is printed. Upon completion, the printed substrate is inspected for defects before being discharged, and the next unprinted substrate is loaded for processing.

Under these conditions, the printing of each substrate must be completed within the production beat time, while ensuring the accuracy of each ejected droplet. In this paper, we denote by $T$ the beat time for a single substrate, which includes the total duration of substrate positioning, deskewing, transferring, and other related operations. We define the non-printing time as $t_{{\text{c}}}$, and the time limit allocated for the printing operation as $t_{{\text{p}}}$, which can be expressed as:

1 $$\mathop \sum \nolimits_{q = 1}^{N} \left( {\frac{{d_{{{\text{y}},q}} }}{{v_{{\text{y}}} }} + \frac{{d_{{{\text{x}},q}} }}{{v_{{\text{x,p}}} }} + \frac{{d_{{{\text{x}},q}} }}{{v_{{\text{x,b}}} }}} \right) = t_{{\text{p}}} \le T - t_{{\text{c}}}$$

where, $N$ represents the total number of printing cycles, referred to as the pass count;$d_{{{\text{y}},q}}$ and $d_{{{\text{x}},q}}$ respectively denote the distance the nozzle moves along the Y-axis and the substrate moves along the X-axis during the $q$-th pass, $q \in \left[ {1,N} \right]$; $v_{{\text{y}}}$,$v_{{\text{x,p}}}$ and $v_{{\text{x,b}}}$ represent the Y-axis movement speed, the X-axis print stroke movement speed, and the X-axis return speed, respectively. Therefore, reducing the number of printing cycles will significantly shorten the required printing time.

Therefore, this study defines three key parameters to evaluate the performance of print planning: solution time, printing time, and printing accuracy. In the in-line printing manufacturing process, printing time is critical due to the constraints imposed by the production beat time. To ensure printing is completed within a stable timeframe, the planning process is typically finished well in advance. Although there are no strict requirements on planning time, achieving faster solutions is clearly advantageous. Printing accuracy directly affects substrate yield, as misaligned ink droplets can severely degrade production quality.

**Fig. 3:**
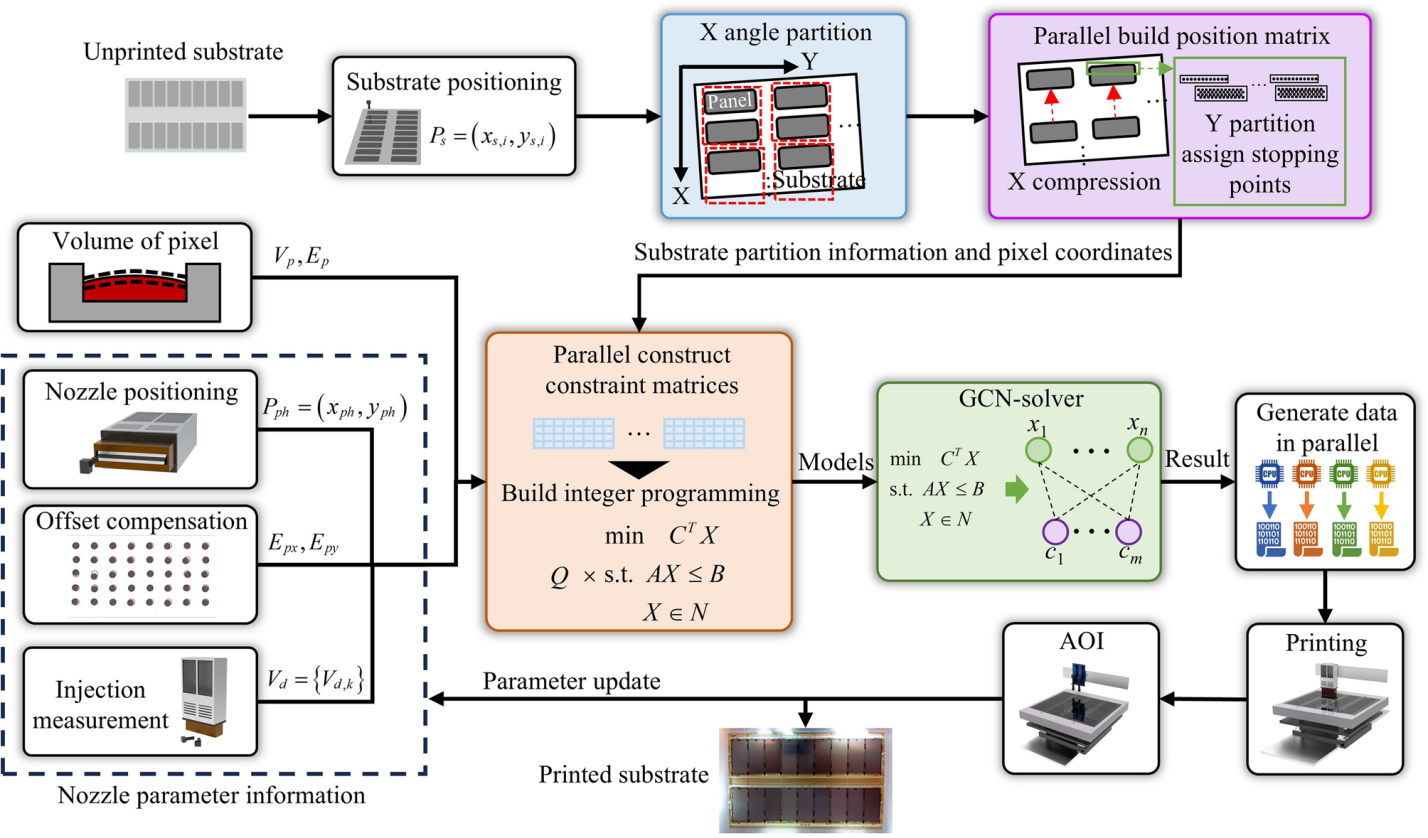
Algorithmic process for large area substrate printing planning based on graph attention networks and integer planning.

However, existing print planning methods are only oriented to small-size substrates and do not take into account the effect of substrate off-angle on printing. The generation of off-angle will seriously affect the efficiency of planning and printing accuracy. In this study, we propose the GIP-LASP, as shown in Fig. 3. The flow represented in Fig. 3 is an experimental flow, and the online production process needs to complete the part of print planning in advance. The unprinted substrate is passed into the printing equipment, and the substrate is first positioned for deskewing. When the upper limit of the equipment deflection angle is reached, the measurement of the substrate deflection angle is started. And based on the deflection angle, it starts partitioning the substrate and assigning stopping points for each partition. This is the position definition at the pixel level. As for the definition of the printing recipe for the whole substrate, it includes the printing volume of the pixel pit and the error range. Also based on the current state of the printhead module, the position coordinates of the nozzles, the drop deviation and the volume are passed into the model building process. After completing the model construction of the partition, all the print data are output in parallel through graph attention network solving. Execute the results of the print planning and output the substrate through AOI defect detection. If there are defects, it is fed back to the printhead status module to update the parameters of the printhead module. This is the general flow of the whole algorithm.

In this study, the integer programming model is designed with the objective of minimizing the number of print actions, thereby reducing the overall printing time. In addition, the solution approach based on imitation learning using a graph attention network (GAT) outperforms the original solver, significantly shortening the planning time. Finally, by modelling the substrate using both X- and Y-directional partitions, the proposed method improves printing accuracy. Although this partitioning strategy may slightly increase the computational burden, it leads to a more precise print path and better print quality.

## Integer programming modelling and GAT-based solution algorithm

The GIP-LASP algorithm proposed in this paper comprises two key components: an integer programming model for inkjet printing and a parallel solution framework based on graph attention networks. First, the integer programming model serves as the foundation of the entire method. It is constructed by defining a set of equally spaced stopping points along the printhead’s motion path, establishing the spatial correspondence between nozzles and pixel pits at each point, formulating jetting constraints, and setting the objective of minimizing the total number of inkjet activations. Second, during model construction, the pixels arranged along the X-direction of the substrate are divided based on the angular deviation, while the Y-direction is partitioned using an optimized segmentation strategy. This results in multiple independent subregions within each projected panel. For each subregion, a separate integer programming model is built. These models are then solved in parallel using a graph attention network trained via imitation learning. To further enhance efficiency, multiple threads are employed to simultaneously generate binary data files for each print region, significantly accelerating the inkjet print planning process.

### Integer programming modelling method for inkjet printing

The primary objective of designing a print integer programming model is to optimize the print path while ensuring that droplets are accurately deposited into their corresponding pixel pits. This requires establishing the spatial relationship between each nozzle and pixel pit. The method for parameterizing the printhead module and pixel pit substrate has been detailed in Section II. Prior to printing, it is essential to evaluate the status of each nozzle, as deviations in nozzle behaviour directly impact both droplet placement accuracy and volume precision. Key droplet characteristics include volume, ejection angle, and velocity. To ensure precise volume filling of the pixel pits, the droplet volume and positional offset at the designated print height for each nozzle must be determined. The average droplet volume measured by the droplet observation system is used as the effective volume input in the planning model. Additionally, during the observation process, the droplet’s ejection angle and velocity are also measured and used as criteria for detecting and filtering abnormal nozzles.

In order to ensure a precise droplet landing point, this study utilizes the offset of the droplet ejected by the nozzle to determine if precise deposition can be achieved in the pixel pit. The injection error $d_{{{\text{error}},k}}$ for the $k$-th nozzle is expressed as:

2 $$d_{{{\text{error}},k}} = h \cdot \tan \theta_{k} - \frac{{v_{{{\text{jet}},k}} \cdot \cos \theta_{k} }}{h} \cdot v_{{{\text{x}},p}}$$

where, $h$ represents the printing height, $\theta_{k}$ is the ejection angle, and $v_{{{\text{jet}},k}}$ is the ejection speed. Some nozzle droplet errors that do not meet the requirements need to be filtered out due to surface strain. To satisfy the compensation for qualified nozzle droplet points, the median of all qualified nozzles is taken as the overall compensation for printing, $d_{{\text{error,m}}}$:

3 $$d_{{\text{error,m}}} = {\text{med}}\left\{ {d_{{{\text{error}},k}} } \right\}_{k = 1}^{m}$$

med is the function to find the median,$m$ represents the total number of qualified nozzles, and the pixel size is set to $R_{{\text{y}}}$. The printable range of pixel $r_{y,j}$ is defined as:

4 $$r_{{{\text{y}},j}} = R_{{\text{y}}} - 2 \times d_{{\text{error,m}}}$$

**Fig. 4:**
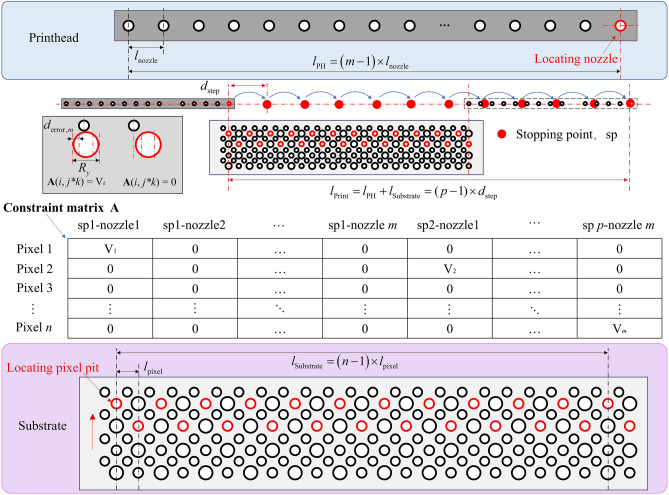
Schematic diagram of the inkjet integer programming modelling approach: parameterize the printhead and substrate, setting *p* equidistant stopping points based on the widths of the printhead and substrate, obtain a constraint matrix defining the relationship between nozzles and pixel pits at each stopping point.

In Fig. 4, we show how to establish the positioning relationship between the pixel pits and all nozzles to create the positional constraints of the model. First, printing takes place along the X direction. Ideally, pixels aligned along the X-direction are ejected by the same set of nozzles, which can be simplified to one pixel participating in the planning process. Therefore the number of pixels is denoted as $n$, which represents the number of pixels in the Y direction.

When establishing the positioning relationship, we align the last nozzle of the printhead with the first pixel pit, move the nozzle evenly along the Y direction for a certain length, and set it as a stopping point. This means that the nozzle can print at these stopping points during the actual printing process. At the final end point, the Y coordinate of the first nozzle of the printhead is greater than the Y coordinate of the last pixel pit, and the number of stopping points is set to $p$. If the absolute value of the difference between the Y-coordinate of nozzle $k$ and pixel pit $i$ is less than the printable range of the pixel pit, then nozzle $k$ can print pixel $i$ at this position and the volume value of this nozzle is recorded as a coefficient. Then the positional relationship matrix between nozzle and pixel pit is as follows:

5 $${\varvec{A}} = \left( {\begin{array}{*{20}c} {a_{1,1} } & {a_{1,2} } & \cdots & {a_{1,m \times p} } \\ {a_{2,1} } & {a_{2,2} } & \cdots & {a_{2,m \times p} } \\ \vdots & \vdots & \ddots & \vdots \\ {a_{n,1} } & {a_{n,2} } & \cdots & {a_{n,m \times p} } \\ \end{array} } \right)$$

6 $$a_{i,k \times j} = \left\{ {\begin{array}{*{20}c} {V_{{{\text{d}},k}} ,\left| {y_{{{\text{p}},ij}} - y_{{{\text{n}},k}} } \right|} \\ {0,{\text{ other}}} \\ \end{array} } \right.$$

where ***A*** ∈ ℝ^*n*×(*m*×*p*)^, and the $i$-th row represents the volume vector for which pixel $i$ can be jetted to all stopping points. After obtaining all possible injection scenarios, a decision needs to be made regarding which volume to select for each pixel $i$. At this point, the decision variable $X$ represents the combination situation for pixel $i$:

7 $${\varvec{X}} = \left( {\begin{array}{*{20}c} {x_{1,1} } \\ {x_{2,1} } \\ \vdots \\ {x_{m \times p,1} } \\ \end{array} } \right)$$

8 $$x_{k \times j,1} \in \left[ {0,N_{{{\text{jet}}}} } \right]$$

where ***X*** ∈ ℤ^*n*×(*m*×*p*)^ represents the number of jetting times at stopping point $j$ for the $k$-th nozzle, $N_{{{\text{jet}}}}$ is the maximum printing times for a single pixel. The integer programming model is formulated as follows:

9 $$\begin{gathered} \min \sum\nolimits_{j = 1}^{p} {y_{j,1} } \hfill \\ {\text{s}}{\text{.t}}{.}\left\{ {\begin{array}{*{20}l} {\sum\nolimits_{k = 1}^{m} {\sum\nolimits_{j = 1}^{p} {a_{i,k \times j} x_{k \times j,1} \le V_{{{\text{ub}}}} ,\forall i \in \left[ {1,n} \right]} } } \hfill \\ {\sum\nolimits_{k = 1}^{m} {\sum\nolimits_{j = 1}^{p} {a_{i,k \times j} x_{k \times j,1} \ge V_{{{\text{lb}}}} ,\forall i \in \left[ {1,n} \right]} } } \hfill \\ {x_{k \times j,1} - y_{j,1} \le 0,\forall k \in \left[ {1,m} \right],\forall j \in \left[ {1,p} \right]} \hfill \\ {x_{k \times j,1} ,y_{j,1} \ge 0,x_{k \times j,1} ,y_{j,1} \le N_{{{\text{jet}}}} ,x_{k \times j,1} ,y_{j,1} \in Z} \hfill \\ \end{array} } \right. \hfill \\ \end{gathered}$$

$V_{{{\text{ub}}}}$ and $V_{{{\text{lb}}}}$ represent the upper and lower bounds for the desired printing volume, respectively. The optimization objective is the minimization of the total printing times, denoted by the sum of $y_{j,1}$, where $y_{j,1}$ represents the maximum printing frequency for all nozzles at stopping point $j$. By solving the integer programming model, the optimal planning model with the minimum printing times under the current conditions can be obtained.

### Integer programming algorithm based on multi-head GAT

**Fig. 5:**
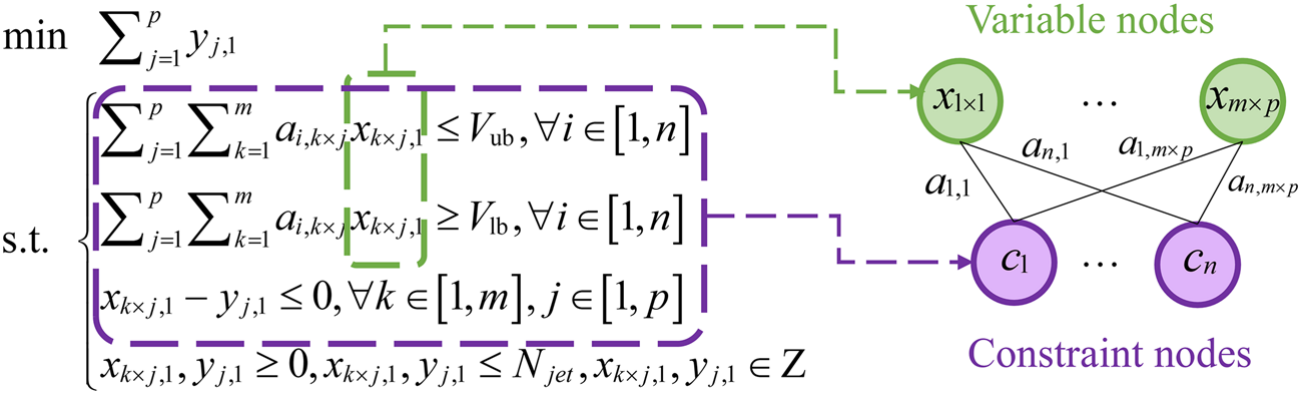
Convert the printing integer programming model into a bipartite graph representation.

We begin by transforming the integer programming model of the print path planning problem into a graph-based structure. According to existing studies, bipartite graphs are the most widely adopted representation, in which variables and constraints are modelled as two distinct sets of nodes. Specifically, an edge $e_{ijk}$ is created between nodes when the coefficient of variable $x_{k \times j}$ in constraint $c_{i}$ is non-zero. Based on this formulation, the printing-related integer programming problem can be represented as a bipartite graph $G = \left\langle {C,E,X} \right\rangle$, as illustrated in Fig. 5, where $C$, $E$ and $X$ denote the sets of constraint nodes, edges, and variable nodes, respectively. To learn an effective branching strategy, we apply imitation learning using GCNs, which sample decisions made by the solver to construct training data. However, conventional GCNs assign uniform weights to all neighbouring nodes, limiting the model’s ability to identify and prioritize critical variables. To overcome this limitation and enhance the network’s representational capacity, we introduce a multi-head GAT into the imitation learning framework. By computing attention coefficients between nodes and dynamically adjusting their weights, the model is better able to focus on the most influential neighbouring variables during training.

First, the network computes the attention coefficients between nodes by sharing weights $\mathop{a}\limits^{\rightharpoonup}$:

10 $$a_{{i,k \times j}} = \frac{{\exp \left( {{\text{LeakyReLU}}\left( {\overset{\lower0.5em\hbox{$\smash{\scriptscriptstyle\rightharpoonup}$}} {a} ^{T} \left[ {Wh_{i} ||Wh_{{k \times j}} } \right]} \right)} \right)}}{{\sum\nolimits_{{l \in N_{i} }} {\exp \left( {{\text{LeakyReLU}}\left( {\overset{\lower0.5em\hbox{$\smash{\scriptscriptstyle\rightharpoonup}$}} {a} ^{T} \left[ {Wh_{i} ||Wh_{l} } \right]} \right)} \right)} }}$$

where, $h_{i}$ and $h_{k \times j}$ are the input features, and $W$ is the linear transformation matrix. The output of multi-head attention is aggregated by the mean to enhance the expression ability:

11 $$h_{i}^{\prime } = \frac{1}{N}\sum\nolimits_{n = 1}^{N} {\sigma \left( {\sum\nolimits_{{l \in N_{i} }} {a_{i,k \times j}^{(n)} W^{\left( n \right)} h_{l} } } \right)}$$

where $\sigma$ is the activation function. After multiple layers are superimposed and passed into the multi-layer perceptron, the branch score $s_{i}$ is obtained, and then the probability distribution of the candidate variables is generated:

12 $$\pi_{\theta } \left( {a^{ * } |s} \right) = \frac{{\exp \left( {s_{{a^{ * } }} } \right)}}{{\sum\nolimits_{a \in V} {\exp \left( {s_{a} } \right)} }}$$

where $\theta$ is the network parameter, and the obtained $\pi_{\theta } \left( {a^{ * } |s} \right)$ is the branching strategy. The problem-solving state of each time sequence of the strong branching strategy and the variable selection action made by the current state are sampled $D = \left\{ {\left( {s_{i} ,a_{i}^{ * } } \right)} \right\}_{i = 1}^{M}$ as the training data set, and the branching strategy is learned by minimizing the cross entropy loss:

13 $$L\left( \theta \right) = - \frac{1}{M}\sum\limits_{{\left( {s,a^{ * } } \right) \in D}} {\log \pi_{\theta } \left( {a^{ * } |s} \right)}$$

**Fig. 6:**
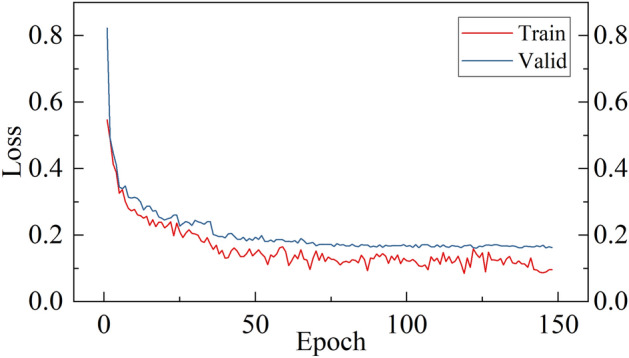
Loss convergence curve of multi-head graph attention network training process.

**Fig. 7:**
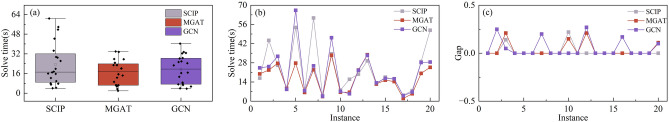
Comparison of the solving results of MGAT, SCIP solver and GCN imitation learning: (**a**) distribution of solving case time; (**b**) solving case time curve; (**c**) solving case gap value.

Based on the above method, we carried out data collection of printing planning data based on SCIP solver, trained the solution algorithm based on multi-head GAT, and the loss curve of the training process and the solution effect comparison with the original SCIP and GCN solution algorithms are shown in Figs. 6 and 7 respectively. The solution algorithm based on multi-head GAT shortens the solution time, and the average Gap value after solution is the lowest, which verifies the superiority of the multi-head GAT network in solution effect.

### Parallel computing framework

As previously described, in an ideal case without substrate skew, pixels along the X-direction can be projected onto a single row, significantly reducing the number of pixel pits involved in planning and thereby shrinking the problem size. However, when the substrate exhibits angular deviation, X-direction partitioning must be applied to realign offset pixels. Notably, each additional X-direction partition effectively doubles the number of pixels involved in planning. Despite this, the Y-direction still contains a substantial number of pixels, posing a major challenge for computation. It is important to note that large-size substrates are composed of multiple small panels arranged in both the X- and Y-directions, which are eventually cut into individual screens. After X-direction partitioning, the resulting increased pixel set is projected onto the first row of panels, effectively creating a new set of Y-direction panels. To reduce the dimensionality of the problem, this study slices the pixel regions within each panel and treats each Y-direction panel as an independent planning unit. An X-direction partitioning strategy is developed based on the droplet deviation and pixel pit size, while the optimal Y-direction partitioning rule is derived via computational simulation. Furthermore, as the substrate size increases, the size and generation time of the corresponding print data files also grow significantly. To address this, a parallel computing strategy is implemented to accelerate the generation of inkjet print data and improve overall planning efficiency.

The X-direction zoning planning scheme is first determined based on the upper limit of the nozzle jetting error and the substrate angle calibration accuracy, to achieve the goal of planning the minimum number of pixel pits for the entire substrate pattern. Assuming that the calibration error angle of the substrate is $\delta$, the allowable deviation distance is determined based on the printable range of the pixel pits. The printable range of the pixel pit is determined by the median of the pixel size and the nozzle jetting deviation, denoted as $d_{{\text{error,m}}}$. The tolerable deviation $l_{{\text{s}}}$ in the X-direction can then be expressed as:

14 $$l_{{\text{s}}} = \frac{{r_{{{\text{y}},j}} }}{\tan \delta }$$

According to the X-direction spacing $g_{{{\text{px}}}}$ of pixels, the number of pixel X -direction lines $n_{{{\text{sx}}}}$ can be obtained as a simplification:

15 $$n_{{{\text{sx}}}} = {\text{floor}} \left( {\frac{{l_{{\text{s}}} }}{{g_{{{\text{px}}}} }}} \right)$$

where, the floor function represents rounding down. Substituting Eqs. (14) and (15) yields the simplified number of pixel rows when the substrate deflection angle is $\delta$, denoted as:

16 $$n_{{{\text{sx}}}} = {\text{floor}} \left( {\frac{{R_{{\text{y}}} - 2 \cdot {\text{median}} \left( {\left[ {d_{{{\text{error}},1}} ,d_{{{\text{error}},2}} , \cdots d_{{{\text{error}},m}} } \right]} \right)}}{{g_{{{\text{px}}}} \tan \delta }}} \right)$$

In accordance with this equation, we can determine the simplified number of divided rows and subsequently partition the entire substrate into regions. Let $N_{{{\text{px}}}}$ represent the total number of rows in the X-direction, $N_{{{\text{py}}}}$ denote the total number of columns in the Y-direction, and $n$ stand for the total number of pixels planned after partitioning:

17 $$n = \frac{{N_{{{\text{px}}}} \cdot N_{{{\text{py}}}} }}{{n_{{{\text{sx}}}} }}$$

Therefore, we simplified the X-direction multi-region partitioning process for the entire large-sized substrate based on the deflection angle, reducing the number of pixels involved in the planning process and thus decreasing the scale of the planning model. Regarding the Y-direction partitioning, no clear rule exists, and we summarize the pattern through computational simulation, which is analysed in detail in Chapter 4. When constructing the model, we set multiple stopping points, where the spacing between stopping points directly affects the number of nozzles that can print within each pixel, influencing both the quality of the optimal solution and the complexity of the problem. Similarly, the number of partitions reduces some constraints and variables from the original large problem, thereby altering the feasible domain and reducing complexity. The modelling approach for the Y-direction partitioning is similar to that for a single panel: first, the number of stopping points $p$ is set, obtaining the set of all stopping points $P$, and each partition receives a subset of $P$. When the coordinates of the printhead at stopping point $j$ satisfy the following inequality, the stopping point is included in the current partition $q$:

18 $$\left\{ \begin{gathered} y_{j\max } \ge y_{q\min } \hfill \\ y_{j\min } \le y_{q\max } \hfill \\ \end{gathered} \right.$$

where $y_{j\max }$ and $y_{j\min }$ are the maximum and minimum Y-coordinates of the nozzle when the printhead is at stopping point $j$, $y_{q\max }$ and $y_{q\min }$ are the maximum and minimum Y-coordinates of the pixel in partition $q$. Therefore, stopping points may be shared between partitions, and the final merged solution will take the maximum value at each stopping point from the corresponding partitions.

In order to efficiently establish the localization relationship between each region and nozzle, this study employs a multi-threaded parallel construction of the nozzle–pixel localization relationship matrix. Each time the nozzle array takes a step along the Y-axis, it is determined whether all nozzles are able to jet at that position, thereby establishing the localization relationship between all nozzles and all pixels. Algorithm 1 is used to determine the spacing between each pixel pit and the nozzle within that region. To reduce the number of evaluations between nozzles and pixels, Algorithm 1 checks only the nozzles near each pixel coordinate. When a nozzle is located within the printable range of a pixel, the corresponding position in matrix *A* is assigned the volume value of that nozzle. Each row of matrix *A* thus represents the volumes of all pixel pits that can be printed by a given nozzle. After the localization relationships are constructed in parallel, the pixel volume constraints involved in the planning process are fully defined. The number of droplets ejected by each nozzle at each stopping point is used as a decision variable, ensuring that the total volume deposited into each pixel remains within a reasonable range. The total number of jets from all nozzles is set as the model’s optimization objective. Since most elements in matrix *A* are zero, storing it as a sparse matrix can significantly reduce memory usage. In this study, the COO format is used to store column indices, row indices, and non-zero element values.

**Algorithm 1:**
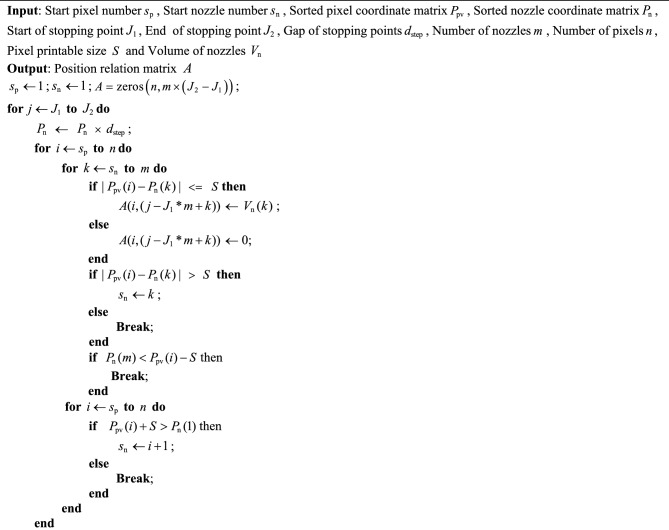
Construct constraint matrix.

After solving the integer planning model using the MGAT-solver, a binary file must be generated to control the printhead during the printing process. Each simplified planning pixel must be expanded across the entire substrate to determine all the pixel pits it represents. The print positions are then specified within a single binary file. Based on the deflection angle of the substrate, the X-direction spacing of the deflected pixels is calculated to generate the X-direction coordinates for all pixels represented by the simplified planning pixels. Additionally, based on the printing resolution, the algorithm identifies the rows in the print data that correspond to each pixel pit’s print point. Data corresponding to nozzle rows is set to 1, while all other data is set to 0. The specific procedure is described in Algorithm 2. The print data is influenced by both the size of the print pattern and the printing resolution. Accordingly, the number of rows in the print data is calculated as the quotient of the print pattern length divided by the resolution, while the number of columns corresponds to the number of nozzles in the Y-direction. When printing on large-size substrates, where the pixel array spans meter-level dimensions and the resolution is at the micro-meter scale, the size of the print data can reach the order of 10^6^. This significantly impacts computational efficiency, making it necessary to store the print data using a sparse matrix representation.

**Algorithm 2:**
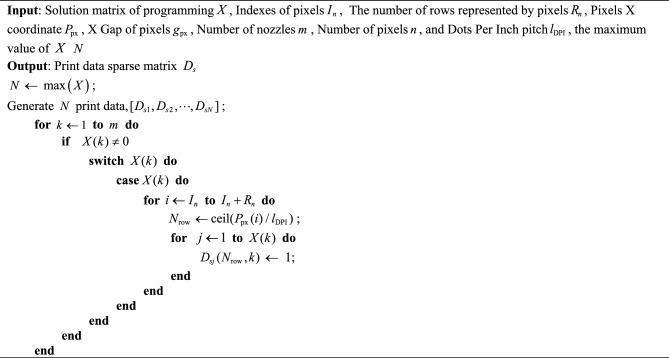
Generate print data.

The print data for each print is stored using a sparse matrix, which is efficiently output to a binary file. Since there is no direct data dependency between different prints, the output process of the print files can be parallelized. The specific process involves converting each sparse matrix into a full print data matrix, and then using multiple threads to generate the binary files containing the print data in parallel.

## Partitioned parallel inkjet printing planning computation simulation

In this section, we analyse the influence of multiple parameters on the solution quality under Y-direction partitioning, and verify the advantage of X-direction partitioning with partitioned planning for large-size substrates. Compared with the integer programming model without Y-direction partitioning, the combined solution obtained by solving multiple subproblems after partitioning may be inferior to the unpartitioned model solution, resulting in a certain gap. This is because partitioned modelling modifies part of the constraints, leading to a shift in the feasible domain and loss of the original optimal solution. However, in practical applications, we aim to strike a balance between solution quality and solving time, so our computational simulations will focus on the overall performance across these metrics. Meanwhile, the need for printing path planning arises from the mismatch between the printhead NPI and the substrate PPI, the nozzle pitch does not match the pixel pit pitch. Therefore, we introduce a spacing ratio parameter $\varphi$, defined as the ratio between the pixel pit spacing and the nozzle spacing, and use it along with the modelled stopping point spacing and the number of partitions as variables in our simulation experiments. We analyse their impact on solution quality and solution time. Finally, we conduct computational experiments involving X- and Y-direction partitioning for G4.5, G6, and G8.5 size substrates, and verify the effectiveness of the proposed zoning scheme by comparing three different print planning methods.

### Multi-parameter computational analysis

**Fig. 8:**
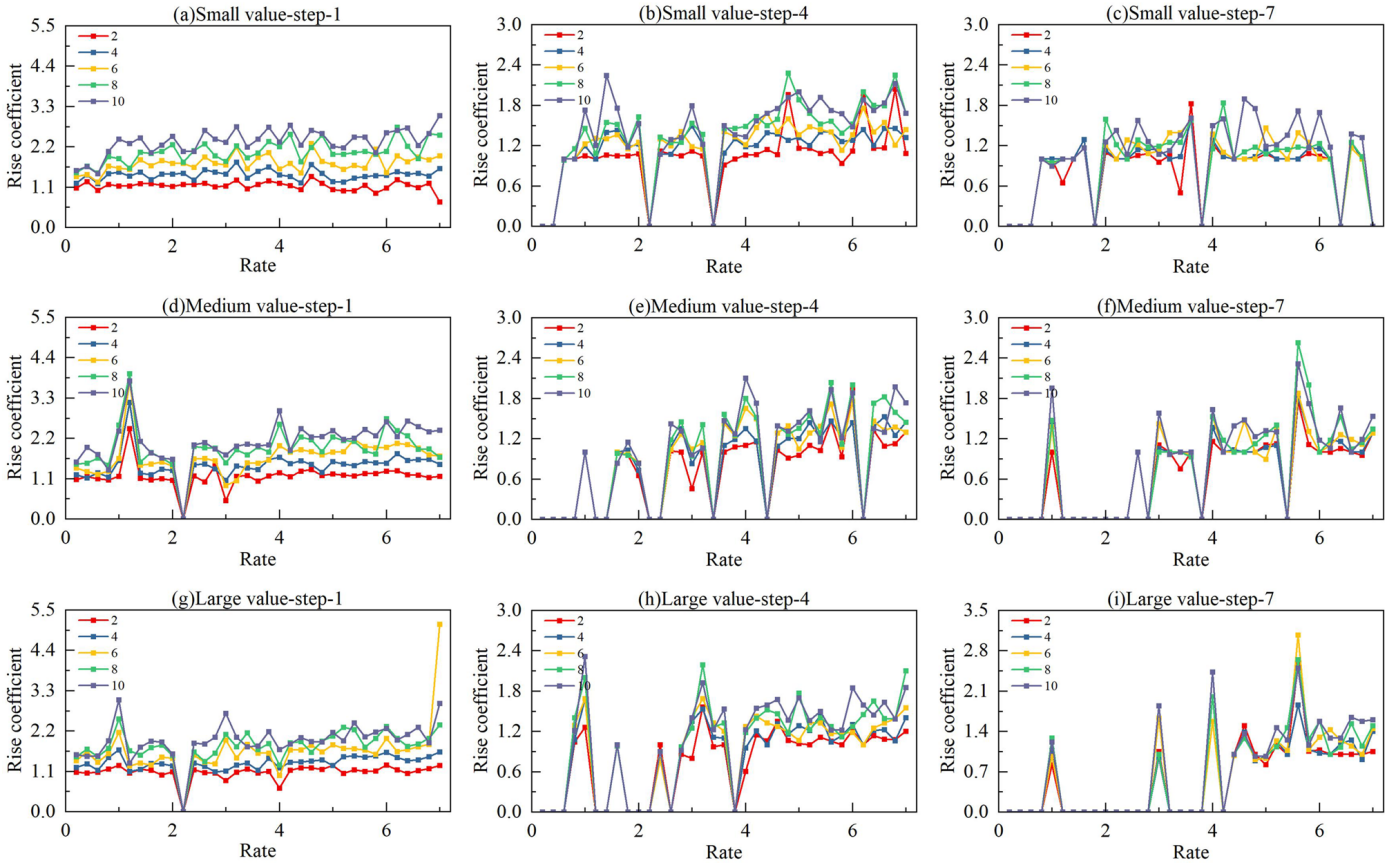
The increment curves of printing times for printing planning problems with 5 partition numbers and 3 stopping point spacings as the ratio of pixel pitch to nozzle pitch increases: (**a**–**c**) small value with steps of 1 mm, 4 mm, and 7 mm; (**d**–**f**) medium value with steps of 1 mm, 4 mm, and 7 mm; (**g**–**i**) large value with steps of 1 mm, 4 mm, and 7 mm.

**Fig. 9:**
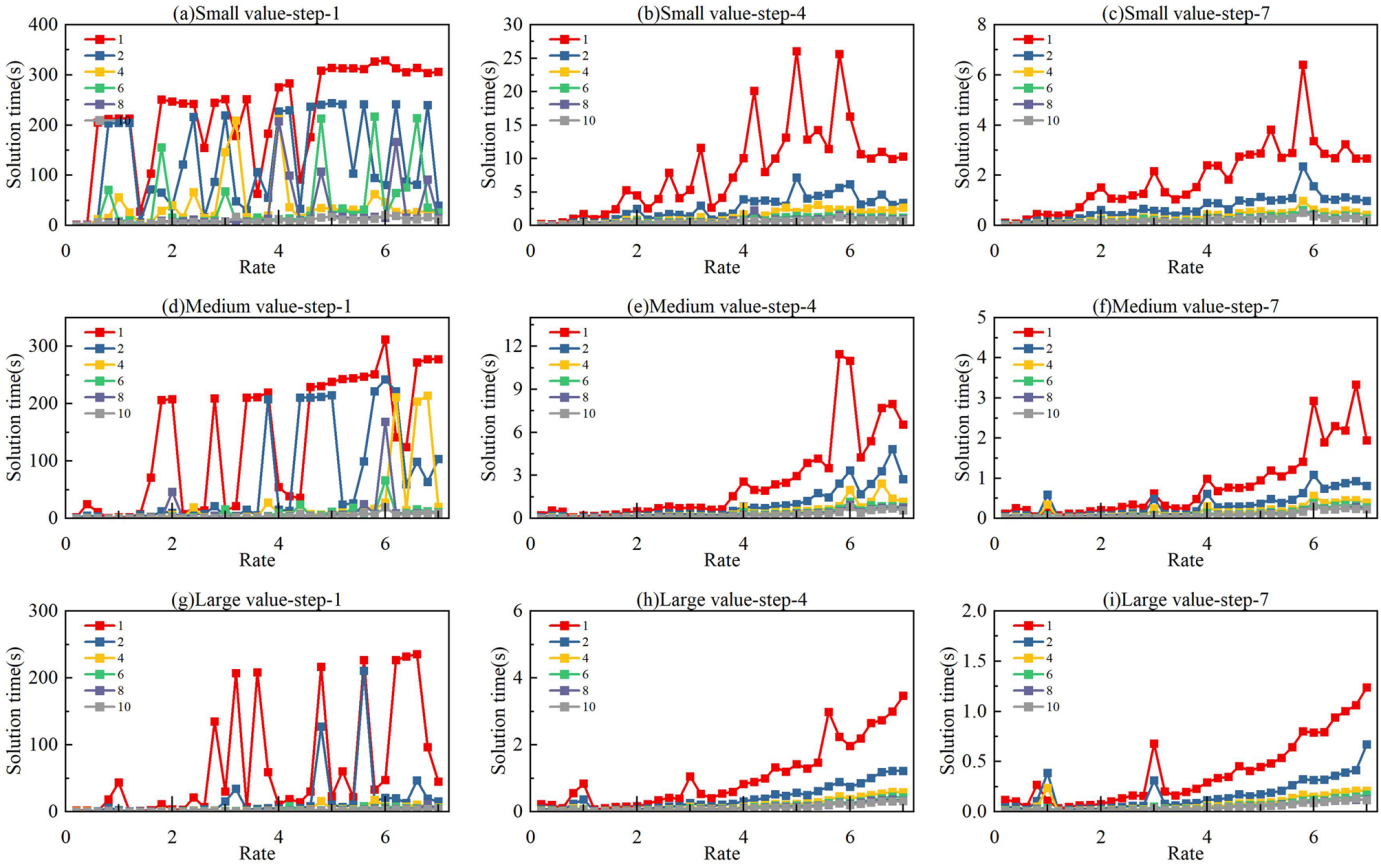
The growth curves of solution time for printing planning problems with 5 partition numbers and 3 stopping point spacings as the ratio of pixel pitch to nozzle pitch increases: (**a**–**c**) small value with steps of 1 mm, 4 mm, and 7 mm; (**d**)-(**f**) medium value with steps of 1 mm, 4 mm, and 7 mm; (**g**–**i**) large value with steps of 1 mm, 4 mm, and 7 mm.

In integer programming modelling, nozzle spacing and pixel spacing are key factors influencing the number of sprayable positions within each pixel. To analyse the impact of different pitch ratios $\varphi$ on both solution quality and solution time, we refer to practical parameter ranges, as summarized in Table 1. Based on specifications from current printhead manufacturers, nozzle resolutions range from 100 to 1200 NPI (nozzle pitch from 254 μm to 21.17 μm), while pixel pit substrates range from 84 PPI for large-screen applications to 460 PPI for smartphone displays (pixel pitch from 300 μm to 55.22 μm). The resulting pitch ratio $\varphi$ thus spans from 0.21 to 14.17. However, when $\varphi$ is relatively large—specifically when a high-NPI printhead is used on a low-PPI substrate—an excessive number of sprayable vias exist within each pixel, significantly increasing the complexity of the integer programming model. Such cases are more suitable for simple printing approaches, such as direct printing, rather than complex planning. Our calculations show that the surplus of sprayable nozzles becomes substantial once the pitch ratio exceeds 7. Therefore, to simplify computation, we set the upper limit of $\varphi$ to 7, cap the maximum solving time at 200 s, and include model construction time when calculating the total solution time. The results are shown in Figs. 8 and 9.

**Table 1:** Parameter range for printed display devices.

Item	Type	Spacing
Min NPI	Konica KM256	254μm
Max NPI	Fuji Samba G3L	21.17μm
Min PPI	Large screen display	300μm
Max PPI	Smartphone screen	55.22μm

To standardize the planning dimensions, we fix both the printhead length and the pixel pit area length., the product of nozzle spacing and the number of nozzles, as well as the product of pixel pit spacing and the number of pixel pits, are set as constants. Since the absolute number of prints cannot be directly compared across planning problems of different scales, we define a rise coefficient as a reference metric for solution quality. The rise coefficient is calculated as the ratio of the number of prints after partitioning to that without partitioning. When the model fails to produce a solution, the rise coefficient is set to 0. We conduct comparative analysis under stopping point spacings of 1 mm, 4 mm, and 7 mm, and vary the number of partitions from 2 to 10 (even numbers only). To account for scenarios where the pitch ratio is the same but the actual values differ, we categorize the settings into small, medium, and large cases. The main independent variables in our study are the pitch ratio (same ratio but with different absolute values: small, medium, large), the number of partitions, and the stopping point spacing (STEP). Through extensive simulation experiments, we analyse how these variables affect two dependent variables: the rise coefficient, which indirectly reflects solution quality, and the solution time.

First, regarding the rise coefficient, a vertical comparison shows that for a fixed stopping point spacing, there is no significant upward trend across the Small, Medium, and Large cases. This indicates that different actual values of nozzle and pixel spacings do not have a substantial impact on the rise coefficient. In contrast, the number of partitions has a clear effect: increasing the number of partitions consistently leads to a higher rise coefficient, implying a decline in solution quality. However, the curves also reveal that when the number of partitions is 2 or 4, the rise coefficient remains close to that of the original unpartitioned model. From a cross-sectional perspective, the tendency for the rise coefficient to increase becomes less pronounced as the stopping point spacing increases. This is because a larger stopping point spacing reduces the solution quality of the original model by decreasing the number of selectable nozzles available for each pixel pit.

As for the solution time, a longitudinal comparison shows that for a fixed stopping point spacing, the Small, Medium, and Large cases show no significant trend differences, nor variations in the order of magnitude. In contrast, the number of partitions has a pronounced impact: more partitions significantly reduce solution time. This improvement is tied to hardware capabilities—stronger parallel computing enhances the trend. However, as partitions increase, hardware limitations impose an upper bound on performance gains. It is evident that when the number of partitions is 2 or 4, the solution time is substantially reduced compared to the unpartitioned model. This confirms that partitioning the model into 2 or 4 segments balances solution accuracy and computational efficiency. In side-by-side comparison, stopping point spacing also has a major influence on solution time. Smaller spacing increases model complexity, thereby lengthening computation time.

**Fig. 10:**
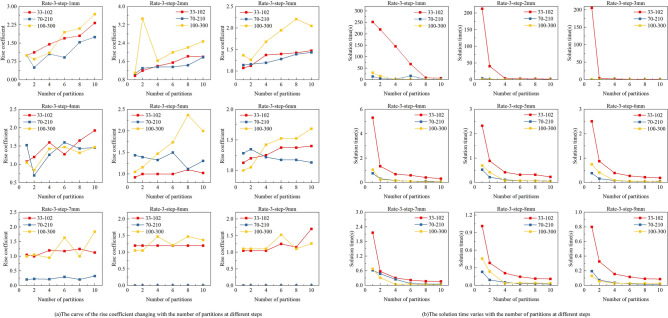
Impact of partition numbers and stopping point spacings on rise coefficient and solving time for spacing ratios 3: (**a**) for rise coefficient; (**b**) for solving time.

**Fig. 11:**
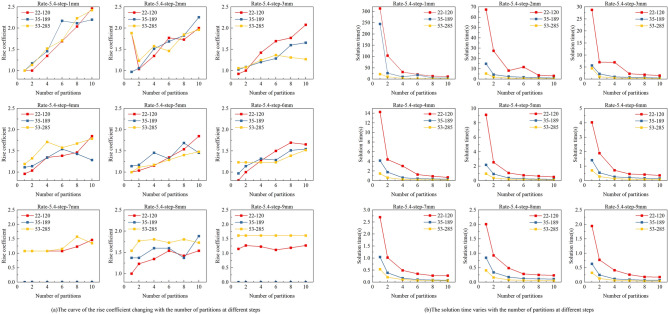
Impact of partition numbers and stopping point spacings on rise coefficient and solving time for spacing ratios 5.4: (**a**) for rise coefficient; (**b**) for solving time.

**Fig. 12:**
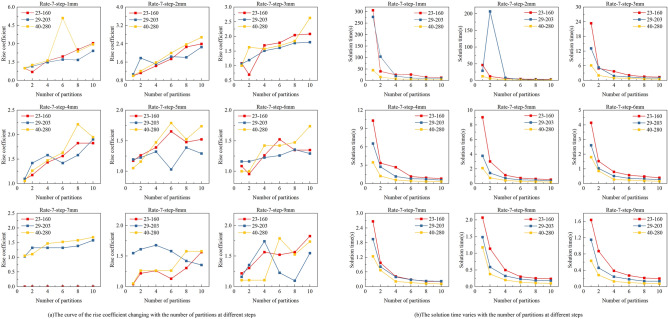
Impact of partition numbers and stopping point spacings on rise coefficient and solving time for spacing ratios 7: (**a**) for rise coefficient; (**b**) for solving time.

Based on the above qualitative analysis, we identified stopping point spacing and the number of partitions as the main factors affecting the performance of the print planning model. We conducted further quantitative analysis to determine guidelines for setting these parameters. To ensure generalizability and avoid randomness, we selected three typical cases with different spacing ratios for comparison, as shown in Figs. 10, 11 and 12.

From the results, the rise coefficient generally increases as the number of partitions grows. However, this increase becomes less pronounced with larger stopping point spacing, since the solution quality of the original (non-partitioned) model also deteriorates in such cases. This suggests that the benefit of partitioning becomes relatively smaller when the base model is already limited in accuracy. In contrast, the solution time shows a significant decline when the number of partitions is set to 2 or 4, regardless of the spacing ratio. For instance, with a ratio of 3 and a stopping point spacing of 2 mm, partitioning into 2 segments leads to a one-order-of-magnitude reduction in solution time. Taken together, these results indicate that setting the number of partitions to 2 or 4 can effectively balance solution accuracy and computation efficiency, making it a practical choice for most planning scenarios.

Based on this analysis, we further determine the appropriate range for selecting the stopping point spacing. When the number of partitions is set to 2 or 4, and in order to ensure solution quality, the rise coefficient for all three ratios continues to increase within the 2–4 mm stopping point spacing range. However, once the spacing exceeds 5 mm, the rise coefficient becomes less sensitive to the number of partitions, and the overall solution quality drops significantly. Therefore, we conclude that the optimal setting is to use 2 or 4 partitions and to keep the stopping point spacing within the range of 2–4 mm.

### Comparison of partition planning effectiveness for large substrates

**Fig. 13:**
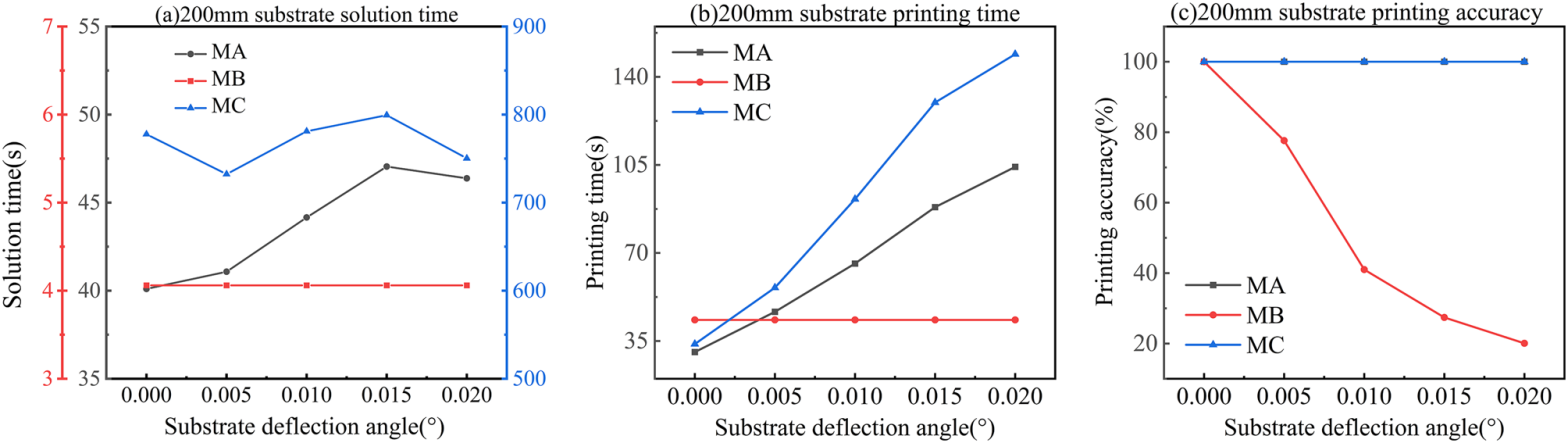
Use MA, MB and MC to calculate 200 mm by 200 mm substrate respectively: (**a**) solution time; (**b**) printing time; (**c**) printing accuracy.

To validate the effectiveness of the GIP-LASP algorithm, we evaluated its performance in terms of solution time, printing time, and printing accuracy. Printing accuracy is defined as the ratio of correctly printed pixel pits to the total number of pixel pits. We compared three methods: Method A (MA), the method proposed in this paper; Method B (MB), which ignores substrate tilt and plans only the first row of pixel pits; and Method C (MC), which plans all pixel pits. The advantages and disadvantages of these three methods were evaluated across different substrate sizes and tilt angles, as shown in Fig. 13.

**Fig. 14:**
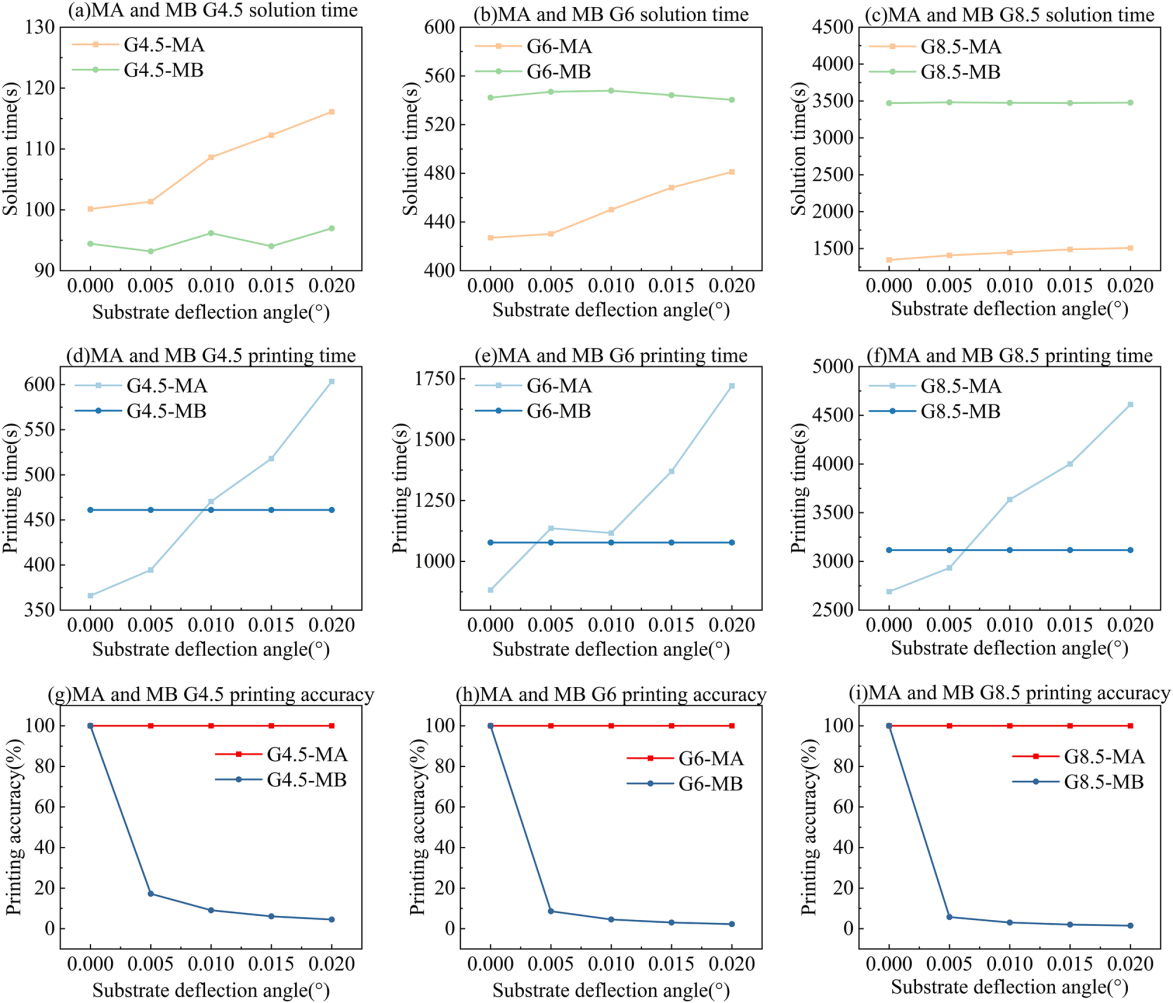
The G4.5, G6 and G8.5 substrates were calculated by MA and MB respectively: (**a**–**c**)G4.5, G6 and G8.5 solution time; (**d**–**f**) G4.5, G6 and G8.5 printing time; (**g**–**i**) G4.5, G6 and G8.5 printing accuracy.

As the substrate tilt angle increases, the planning time for all three methods remains relatively stable; however, MC requires significantly more time than MA and MB. Since MB does not take substrate tilt into account during planning, its printing accuracy decreases as the tilt angle increases, resulting in lower yield rates. In contrast, both MA and MC can maintain 100% printing accuracy. Since each pixel pit needs to be planned separately, the MC method stores the coordinates of all pixel pits in memory when dealing with large substrates. Large substrates have hundreds of millions of pixels, which puts enormous pressure on computer storage. In addition, different substrate angles require a large number of position coordinate updates. Furthermore, during planning, it takes a long time to determine the positional coordinate relationship between the nozzle and all pixel pits through a loop. Similarly, during dynamic planning decisions, the disadvantages of planning too many pixel pits become even more evident. Compared to MA and MB, the difference is tens of orders of magnitude, so MC takes significantly longer than MA and MB in terms of planning time. The computing equipment used in this study cannot withstand this level of computing pressure, so currently, simulation can only be performed on a 200 × 200 mm substrate. Therefore, this paper only discusses the MA and MB methods on large substrates. We calculated the solution time, printing time, and printing accuracy for G4.5 (920 × 730 mm), G6 (1800 × 1500 mm), and G8.5 (2500 × 2200 mm) panel sizes, as shown in Fig. 14.

The planning results using the MA method show that solution time gradually increases with substrate size and tilt angle, but the growth remains within an acceptable range. In contrast, MB exhibits an exponential increase in solution time. The core of the MA method is the relocation of a portion of the pixel pits to ensure accurate printing, which, although it adds complexity to the planning, minimizes the number of relocations. In terms of printing time, since MB does not account for substrate tilt, its printing time remains constant. However, for MA, increased substrate declination leads to longer printing time, which is an expected outcome. Nevertheless, printing accuracy is a critical requirement in industrial production. Unlike MB, the MA method guarantees high printing accuracy and thereby ensures product quality and compliance—a crucial advantage in practical manufacturing scenarios.

## Printing experiment

**Fig. 15:**
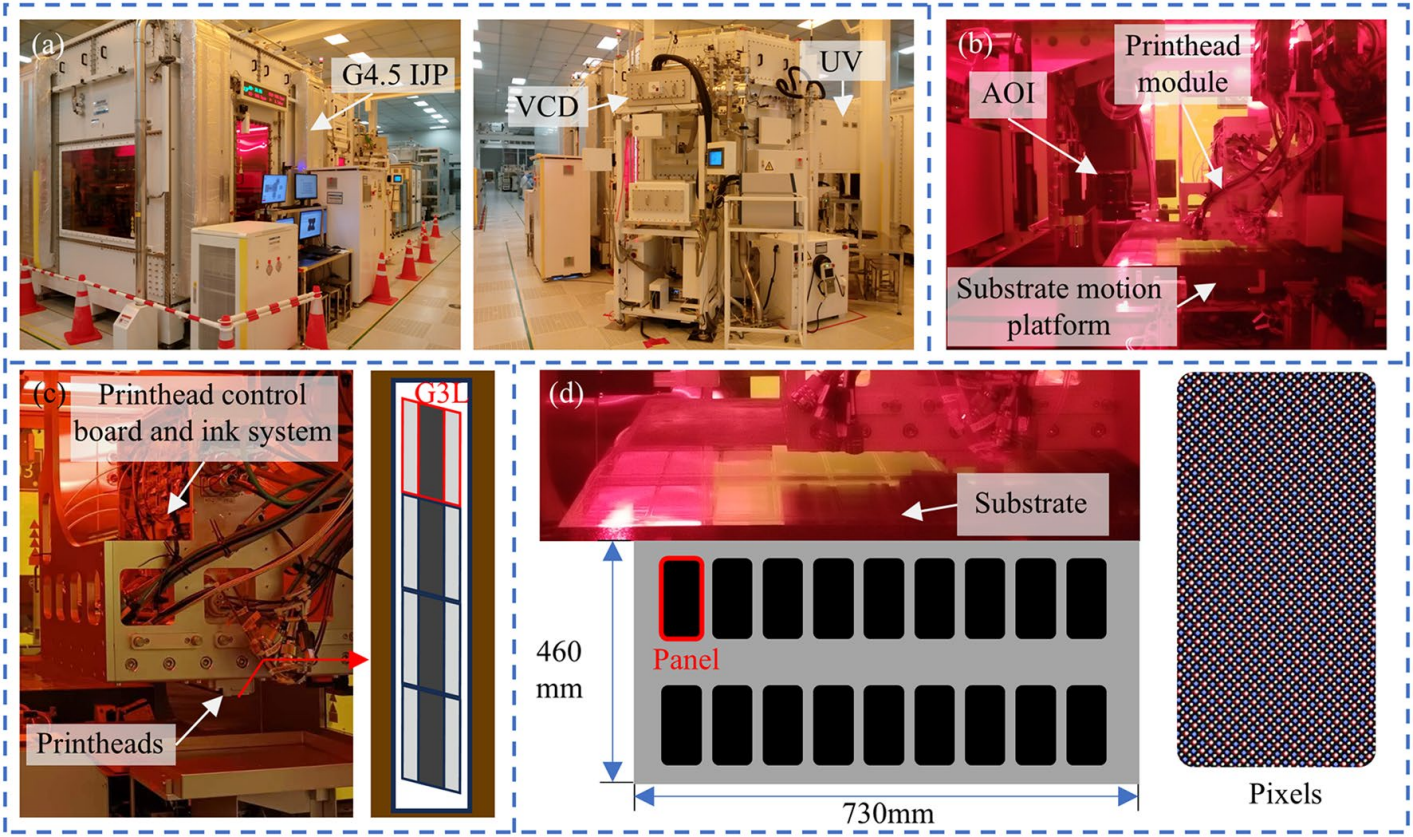
G4.5 high-resolution printing equipment: (**a**) IJP, VCD and other external auxiliary equipment; (**b**) IJP internal structure; (**c**) nozzle module components; (**d**) G4.5 half substrate information.

Based on the planning results of MA and MB, we conducted experimental validation using a G4.5 high-resolution printing platform, jointly developed by the School of Mechanical Science and Engineering at Huazhong University of Science and Technology and Wuhan National Innovation Technology Photoelectric Equipment Co., Ltd. The experimental setup, shown in Fig. 15a, includes components such as a printer, a high-precision robotic handling arm, a UV curing device, and a visual control display (VCD). The internal system, illustrated in Fig. 15b, mainly consists of a droplet volume observation module, a printhead module (moving along the Y-axis), a substrate motion platform (moving along the X-axis), an automatic optical inspection (AOI) unit, and a GIS control board (integrated within the printhead module). The detailed structure of the printhead module is shown in Fig. 15c. This module is assembled from four Fuji Samba G3L printheads, each with 2048 nozzles arranged at an angle. The resolution of the printheads was set to 1200 NPI. Figure 15d shows the substrate used in our experimental prints. It is a half-size G4.5 substrate with dimensions of 730 × 460 mm and a pixel resolution of 394 PPI. A total of 18 panels are arranged on the substrate in a matrix layout. Each sub-pixel measures between 20 and 30 μm.

**Fig. 16:**
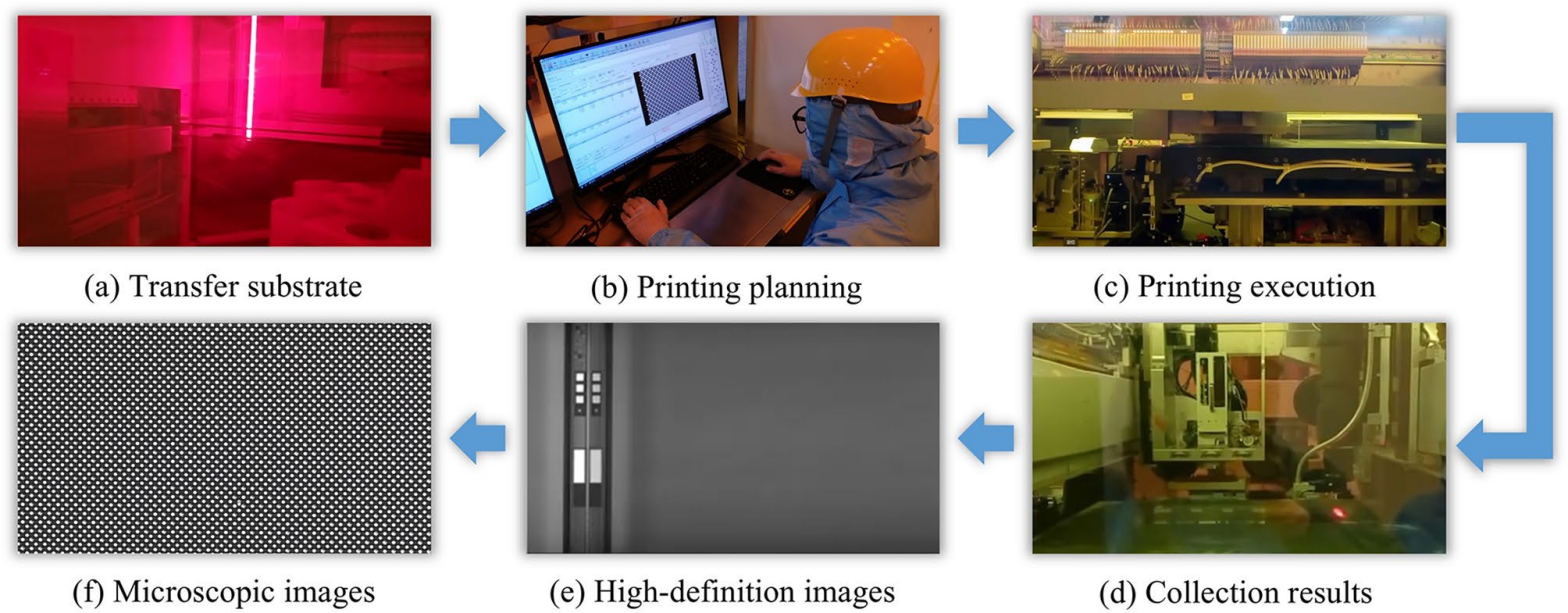
Printing experiment execution process.

The specific experimental workflow is illustrated in Fig. 16, and consists of the following steps: (1) Transferring the substrate to the printer via a robotic arm. (2) Setting parameters and planning the print path (this step is pre-configured in actual production). (3) Executing the printing process. (4) acquiring images and analysing the printing results.

In order to achieve printing efficiency, the concatenation of multiple printheads is employed to extend the printable width in a single printing pass. Based on the planning results, the printhead achieves precise positioning in the Y-direction, transmitting the binary file of each pass to the printhead controller for interpretation and executing the printing process. Ink droplets undergo processes such as ejection, flight, and deposition, ultimately landing within pixel pits. The printing results are captured using a line-scan camera, which enables efficient and comprehensive imaging of the entire substrate surface.

**Fig. 17:**
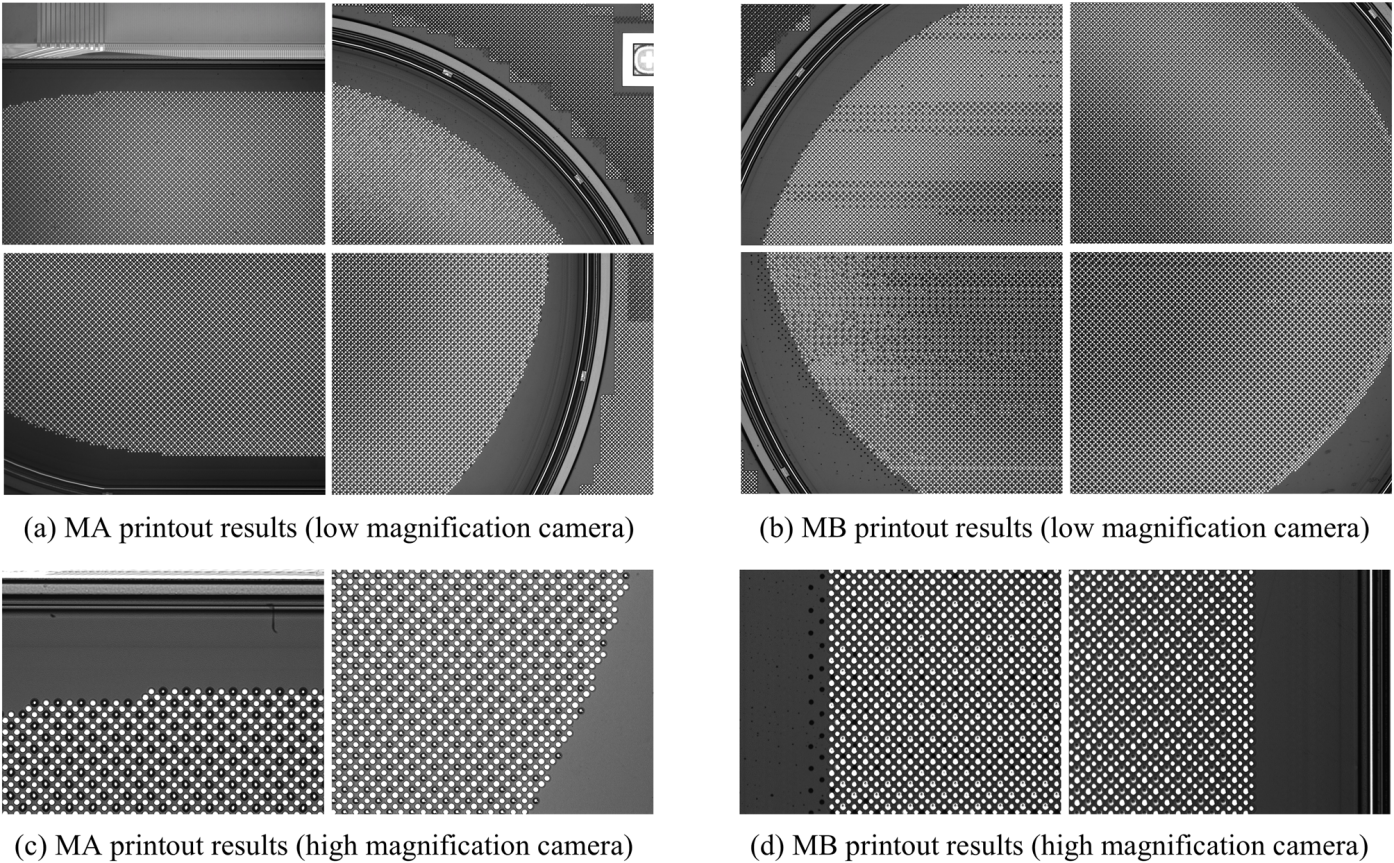
Comparison of MA and MB printing results: (**a**) and (**b**) are the four corners of the substrate printed by MA and MB under low-magnification cameras; (**c**) and (**d**) are the printing results of MA and MB under high-magnification cameras.

We compared MA and MB through actual printing experiments. While the MC method achieves extremely high accuracy by planning every pixel pit in detail, it requires an impractically long planning time, making it unsuitable for real-world production. As shown in Fig. 17, the MA method improves printing accuracy by adaptively adjusting the number of partitions based on the angular deviation of the substrate. In contrast, the MB method leads to noticeable droplet misalignment and tilting, with a clear angular deviation between the printed result and the actual pixel layout on the substrate—failing to meet practical production requirements.

**Fig. 18:**
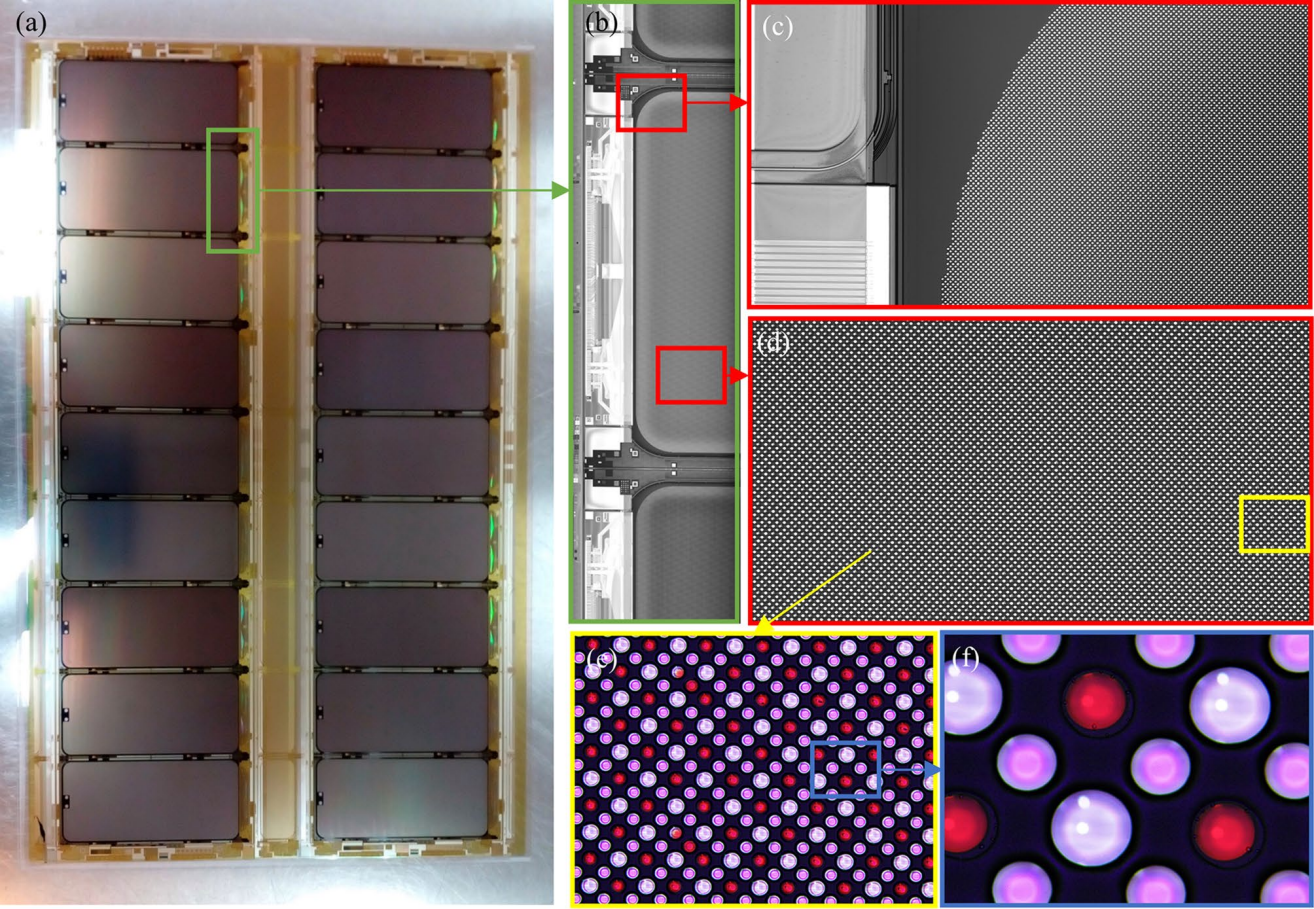
394 PPI G4.5half substrate R pixel printing experiment: (**a**) macroscopic overall view of the substrate; (**b**) AOI line array camera mapping results; (**c**) and (**d**) pictures taken by the camera in the printing equipment; (**e**) and (**f**) high-power microscope inspection of the shooting results.

Based on our proposed GIP_LASP method, we successfully printed a G4.5 half-size substrate, and the actual printing results are shown in Fig. 18. Figure 18a presents the macroscopic overall appearance of the substrate after printing, while Fig. 18b shows the AOI line array camera’s captured results. Figure 18c and d were taken using a camera integrated within the printing rig, equipped with a coaxial light source for substrate illumination and imaging. Figure 18e and Fig. 18f were captured with a high-powered microscope outside the printing equipment. Under the microscope’s illumination, the pixel pits printed with red color filter (CF) ink appear red, while the other pits correspond to the green (G) and blue (B) sub-pixels that were not printed with red ink. All panels were printed simultaneously, and the red pixel illumination on the backlit substrate was successfully achieved. During the experiment, the substrate angular deviation was 0.043°, and the entire panel was free of defects.

## Conclusion

This study analyses the print planning bottlenecks faced in OLED manufacturing during the transition to large-size substrates. The core issue lies in the amplification effect of angular deviation, which significantly increases the number of pixel pits involved in the planning process, thereby affecting both efficiency and planning time. To address this challenge, the proposed solution strategy focuses on two main aspects: (1) reducing the number of pixels involved in the planning, and (2) enhancing solution efficiency through parallelization.

To this end, we propose the GIP-LASP algorithm. For problem (1), we construct a pixel partitioning model based on the angular deviation, treating some pixels as equivalent in the planning process (i.e., printed using the same nozzle). For problem (2), we introduce parallel processing across multiple tasks. First, when constructing the positional relationship between the printhead and the pixels, we parallelize the operation matrix based on the initial pixel partitioning. Each sub-region is then solved using a solution algorithm based on a multi-head map attention neural network. After obtaining the motion path of the printhead, binary files are efficiently stored through multithreading, further improving data generation speed. The main innovations of this study are as follows: (1) The introduction of a parallel computational framework for inkjet print planning on large-size substrates, including an X-direction pixel partitioning rule and a Y-optimal partitioning rule. (2) A method for constructing an inkjet integer planning model that minimizes the number of inkjets while ensuring the ink volume per pixel pit remains within allowable tolerances. (3) The development of a GAT-based integer programming solution algorithm incorporating a multi-head attention mechanism, which demonstrates superior solution efficiency compared to the original SCIP solver in the context of printed display planning. (4) Experimental validation of the proposed method, achieving high-resolution (394 PPI) printing on a G4.5 half-size substrate. The proposed method is also applicable to the printing of HIL, HTL, and TFE layers, and can be extended to QLED technologies, providing an effective solution for large-panel manufacturing and contributing to overall process optimization.

Of course, although this study has achieved effective results, there remains room for further optimization. For instance: (1) The model-solving algorithm could be enhanced by incorporating learning-based approaches such as graph neural networks. (2) The pixel partitioning rules may require further refinement to accommodate higher-resolution demands. (3) Even with simplifications, the computational load remains substantial for larger-size manufacturing, potentially limiting the method’s applicability—therefore, more adaptive and scalable computational strategies should be developed.

## Supplementary Information

Supplementary Information 1.

Supplementary Information 2.

## Data Availability

All data generated or analysed during this study are included in this published article.
