# Supplementary figures and images for: Large-area OLED substrate printing path planning method based on multi-head GAT imitation learning to solve partitioned integer programming
Source: Sci Rep. 2025 Jul 1;15:21771. doi: 10.1038/s41598-025-08355-x (PMC12219173; doi:10.1038/s41598-025-08355-x)

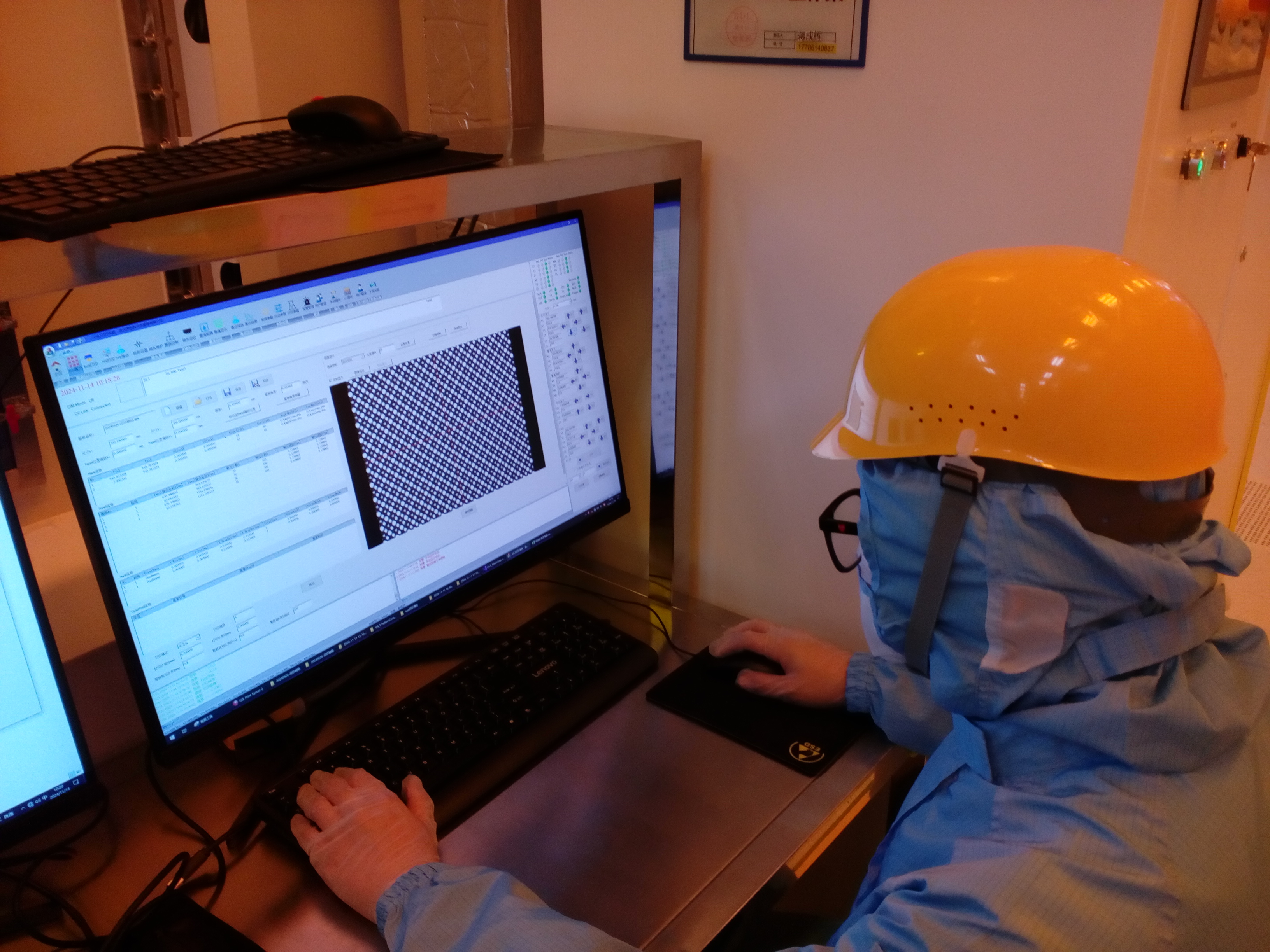

Supplement: Supplementary file 1 — Supplementary Information 1. [file 41598_2025_8355_MOESM1_ESM.zip › Experimental_result_videos_1/2_In operation.jpg]
